# Current Status and Challenges Associated with Tick-Borne Pathogens and Diseases: Where Do We Stand?

**DOI:** 10.3390/pathogens12101271

**Published:** 2023-10-23

**Authors:** Pavle Banović, Islay Rodríguez, Dejan Jakimovski

**Affiliations:** 1 Clinic for Lyme Borreliosis and Other Tick-Borne Diseases, Pasteur Institute Novi Sad, 21000 Novi Sad, Serbia; 2 Department of Microbiology with Parasitology and Immunology, Faculty of Medicine in Novi Sad, University of Novi Sad, 21000 Novi Sad, Serbia; 3 National Reference Laboratory of Treponemes and Special Pathogens, Tropical Medicine Institute “Pedro Kourí”, Havana 10400, Cuba; islay@ipk.sld.cu; 4 Faculty of Medicine, Ss. Cyril and Methodius University in Skopje, 1000 Skopje, North Macedonia; 5 University Clinic for Infectious Diseases and Febrile Conditions, 1000 Skopje, North Macedonia

Lyme Borreliosis (LB), caused by *Borrelia burgdorferi* sensu lato (s.l.) and transmitted by specific *Ixodes* spp. ticks, is the most common vector-borne disease in the United States and the most common tick-borne disease in the northern hemisphere [1,2]. Despite accepted guidelines for diagnosing LB, with specific clinical case definitions, in the absence of relevant clinical information or when faced with an atypical presentation, clinicians tend to rely on serological tests when including LB in the differential diagnosis. Serological tests for LB, conducted in accordance with the European Concerted Action on LB guidelines as part of a two-stage diagnostic process, frequently pose challenges in interpretation, especially with nonspecific clinical presentations. This is primarily attributed to the significant occurrence of false-positive results, which can be influenced by cross-reactivity with acute viral infections [3]. In this Special Issue, Wojciechowska-Koszko et al. [4] confirm that serological tests used in the diagnosis of LB can generate false-positive results in patients with acute viral infections. More precisely, tests used for the first step of the two-stage approach, such as enzyme-linked immunosorbent (ELISA) or indirect immunofluorescence (IIFT), and the immunoblot (IB) method for the second step of the two-stage diagnostic approach all showed significant cross-reactivity and positive results in patients with Epstein–Barr virus (EBV), cytomegalovirus (CMV), and BK virus (BKV) infections without clinical manifestations related to LB.

While *Borrelia burgdorferi* s.l. represents the leading bacterial entity among tick-borne pathogens (TBPs) responsible for causing LB, tick-borne encephalitidis virus (TBEV; *Orthoflavivirus encephalitidis*) stands out as the most important viral pathogen transmitted by hard ticks. Jakimovski et al. [5] address the public health threats of LB and tick-borne encephalitis (TBE) in North Macedonia and Serbia through serological screening of tick-infested individuals for anti-*Borrelia* and TBEV-neutralizing antibodies. The study’s findings show no TBEV-neutralizing antibodies in participants from northern Serbia but a single case with moderate TBEV neutralization in North Macedonia, suggesting the possibility that a TBEV-supporting ecosystem exists in North Macedonia. The study also highlights a greater prevalence of anti-*Borrelia* antibodies in Serbian tick-infested individuals compared to those in North Macedonia. Although the study acknowledges limitations, such as a relatively small sample size, it is considered significant as the first report suggesting possible exposure to members of the *B. burgdorferi* s.l. and/or TBEV in the territory of North Macedonia. It represents a foundational reference for further risk assessment and surveillance of LB and TBE emergence in North Macedonia and Serbia.

Ticks are not uniform in their vector capabilities; their competence to acquire and transmit pathogens varies based on factors such as their species and geographical location. Ticks belonging to the *Hyalomma* genus are known to transmit a wide array of pathogens responsible for diseases affecting both humans and animals. These ticks have been expanding their geographical range in certain regions, which is concerning due to their ability to feed on a diverse range of hosts, including humans. Bonnet et al. [6] conducted a bibliographical study to investigate and provide validation evidence focusing on the vector potential of ticks of the *Hyalomma* genus. While the study provides a list of validated pathogens that can be transmitted via tick bites from *Hyalomma* ticks and highlights a plethora of pathogens potentially transmitted by them, it also emphasizes the need for rigorous experimental validation of vector competence, which is often challenging due to the complexity of tick–host–pathogen interactions.

Although not the most prevalent tick-borne disease, Crimean–Congo hemorrhagic fever (CCHF) is one of the most concerning due to its severe and often fatal consequences in affected individuals, as well as its potential for pandemic spread under certain circumstances [7]. CCHF virus is widespread across Europe, Asia, and Africa [8,9,10]; however, it has never been reported in Australia and the Americas. In this Special Issue, Sultankulova et al. [11] contribute to a deeper understanding of the epidemiology of CCHF by addressing the genetic diversity and prevalence of CCHF virus in the endemic areas of Kazakhstan. Out of 694 collected and analyzed ticks from 3 endemic areas (i.e., Zhambyl, Kyzylorda, and Turkestan), CCHF virus RNA was detected in 4 samples. More precisely, CCHF virus RNA was found in three *Hyalomma asiaticum* ticks and one *I. ricinus* tick. This is the first detection of CCHF virus in *I. ricinus* from Kazakhstan, which gives reason to suspect that this tick species can be involved in the formation and maintenance of a natural focus of CCHF virus. Genetic research on the CCHF virus S segment showed that the virus isolated in Zhambyl and Turkestan belongs to the Asia 2 genotype, and the virus isolated in the Kyzylorda region belongs to the Asia 1 genotype. The study complements previous knowledge regarding the circulation of CCHF virus genotype Asia 2 in the Kyzylorda region by also confirming the presence of the CCHFV Asia 1 genotype in the same region. Furthermore, it demonstrates the circulation of the CCHF virus Asia 2 genetic lineage in the Zhambyl region for the first time.

TBPs affecting both humans and animals globally exhibit a wide range of varieties. Among them are protozoans belonging to the genus *Babesia*, which infect the host’s erythrocytes and give rise to various clinical manifestations. In recent years, the number of babesiosis incidents in Poland and other Central European countries has increased [12]. The prevalence of *Babesia canis* is closely associated with the geographic distribution of the ornate dog tick, *Dermacentor reticulatus*, which serves as the primary carrier for this blood parasite. Previous scientific reports confirmed an area in Poland known as the “gap zone”, which is free of *D. reticulatus* ticks and spans from West Pomerania and Pomerania Voivodeships in Northern Poland to Opole, Silesia, Lesser Poland, and Subcarpathia Voivodeships in the Southern part of the country. In this region, Pawełczyk et al. [13] confirm two cases of *B. canis* infection in domestic dogs with no travel history. Additionally, this study also confirms *D. reticulatus* tick infestation in *Babesia*-infected dogs with no travel history in the previous 8 weeks. These findings should raise awareness among clinicians in the region of Silesian Voivodeship about *B. canis* infection, which should be taken into account during the differential diagnosis of tick-borne diseases.

In the article dealing with TBP spillover, Cumbie et al. reported the discovery of Bourbon virus (BRBV) in ticks in Virginia, USA, and its implications for public health [14]. BRBV is a tick-borne virus that was first identified in Kansas in 2014 and has since been associated with human infections [15]. Their study aimed to screen local *Haemaphysalis longicornis* ticks, an invasive tick species, for BRBV and Heartland virus (HRTV), another tick-borne virus of medical importance. The study was conducted in Virginia, which is home to several tick species, including the invasive *H. longicornis*, an Asian longhorned tick. *H. longicornis* has been found in multiple states in the eastern USA and is a vector for various pathogens [16].

Bourbon virus is a type of Thogotovirus, a segmented, negative-sense RNA virus transmitted by arthropods, including ticks. Before 2014, only Dhori virus and Thogoto virus were known as human pathogens in this group (14). The discovery of BRBV in Kansas marked the first case of human pathogenic Thogotovirus infection in North America. Since 2014, there have been two additional cases of human BRBV infections in the USA, one in Oklahoma and another fatal case in Missouri in 2017. While the virus was initially identified in Kansas, it has since been detected in other states [17,18]. Another tick-borne virus of concern in this article is Heartland virus, which was first identified in Missouri in 2009. It can cause severe symptoms in infected individuals and has been associated with fatalities [19].

Cumbie et al. detected BRBV in *H. longicornis* ticks from multiple counties in western Virginia. While HRTV was not detected, further research is needed to understand its presence in the region. The study also tested the blood of local wildlife, such as white-tailed deer, raccoons, groundhogs, striped skunks, and eastern cottontails, for evidence of exposure to BRBV. Some of these animals showed neutralizing antibodies against BRBV. Their findings indicate that BRBV is circulating in multiple counties in western Virginia, and the presence of BRBV-infected *H. longicornis* ticks raises concerns about its potential spread and impact on public health.

On the other hand, Fang et al. investigated the prevalence of intracellular bacterial pathogens in *Haemaphysalis flava* ticks collected from hedgehogs in Hubei Province, China [20]. The study aimed to identify the presence of bacteria such as *Rickettsia*, *Ehrlichia*, *Anaplasma*, and *Coxiella burnetii* within these ticks, as these bacteria are known to cause severe human diseases and are transmitted by ticks [20]. Tick-borne diseases are prevalent in many parts of China due to its vast territory, complex geography, and different climates. Hard body ticks, including *H. flava*, play an important role in transmitting TBPs. The study identified the presence of *Rickettsia* (*R. japonica* and *R. raoultii*), a novel *Ehrlichia* species, *Coxiella burnetii*, and *Anaplasma bovis* in the collected *H. flava* ticks. The infection rates of these pathogens varied among different tick developmental stages, with dead engorged ticks having the highest TBP prevalence.

An interesting observation by the authors is that the significantly higher infection rate of intracellular bacteria in dead engorged ticks could be attributed to the detrimental effects of the bacteria on ticks or the loss of bacteria during oviposition and molting. This might explain the lower bacteria prevalence in molted adult ticks and eggs.

Tully et al. primarily focus on comparing the infection, persistence, and replication of *Francisella tularensis* in three different tick species (i.e., *Dermacentor variabilis*, *Amblyomma americanum*, and *H. longicornis*). The study directly compared *F. tularensis* infections in *D. variabilis*, *A. americanum*, and *H. longicornis* ticks using a mouse–tick–*F. tularensis* infection model [21]. The study examined the infection, persistence, replication, and transmission of *F. tularensis* in these tick species at different infectious doses. *Francisella tularensis* was found to infect and persist in both *D. variabilis* and *A. americanum* ticks, but *H. longicornis* ticks were unable to support *F. tularensis* infection, suggesting that they are unlikely to be major vectors for tularemia. Although *A. americanum* ticks initially acquired higher *F. tularensis* numbers, in this case, *F. tularensis* replicated more robustly in *D. variabilis* ticks, especially in the first few weeks. The study identified FTL1793, a chitinase enzyme, as a factor contributing to *F. tularensis* persistence and replication in ticks. Chitinases are thought to help *F. tularensis* degrade chitin, a major component of ticks.

Mkize et al. provided a review dealing with the importance of identifying genetic markers associated with tick resistance in cattle through genome-wide association studies (GWAS). They highlighted the economic and environmental significance of tick resistance, the limitations of traditional tick control methods, the role of genotyping technologies and SNPs, and the challenges and opportunities in conducting GWAS for tick resistance [22].

Authors emphasize the need for standardized phenotyping procedures, genotype imputation, collaboration, and the establishment of genetic evaluation programs. It also touches on the potential benefits of genetic selection for tick resistance in cattle breeding programs. It is clear that genetic approaches, such as GWAS, have the potential to improve tick resistance in cattle, reducing the need for chemical control methods that have limitations and can impact both animal health and food safety. Genomic selection, based on the identification of relevant genetic markers, offers a promising avenue for enhancing tick resistance in cattle populations, thereby improving animal health, welfare, and productivity.

Tawana et al.’s paper discusses the prevalence of TBPs in domestic ruminants in the Southern African Development Community (SADC) region. Authors highlight that ticks are a significant concern in the SADC region due to their role as vectors of TBPs, which can affect the health of domestic animals and humans. The SADC region has various tick species belonging to different families, including Argasidae, Ixodidae, and Nuttalliellidae [23].

TBPs are a concern in tropical and subtropical regions of Africa, and their prevalence varies by geographic location, depending on the tick species present [24]. The study conducted a systematic review and meta-analysis of articles reporting tick abundance, TBP prevalence, and distribution in domestic ruminants across the SADC region. The results of the analysis showed that the overall pooled prevalence estimate of TBPs in domestic animals in the SADC region was 52.2%. Cattle had the highest prevalence at 51.2%, followed by sheep (45.4%) and goats (29.9%).

Various TBPs, including *Anaplasma* spp., *Babesia* spp., *Ehrlichia* spp., *Rickettsia* spp., and *Theileria* spp., were documented as significant pathogens in domestic ruminants, particularly cattle [25,26,27,28,29,30]. The review also examined the prevalence of TBPs in different tick species. It found that *Amblyomma* ticks had a higher prevalence of TBPs compared to *Rhipicephalus* ticks, with *Amblyomma variegatum* being the most infected tick species [23].

Authors observed a declining trend in TBP prevalence over the years, suggesting potential improvements in tick and TBP control measures in the region. The study acknowledges limitations, including variations in data availability across SADC countries and differences in study design and sample sizes.

Authors provide insights into the prevalence and distribution of TBPs in domestic ruminants across the SADC region, highlighting the importance of understanding and managing tick-borne diseases in this area. It also suggests the need for further research and collaboration to address this veterinary and public health concern effectively.
